# Modulation of Patient-Derived Tumor Organoids by SARS-CoV-2 Variants Across Cancer Types: A Study Combining Morphology, Inflammation, and Whole-Exome Profiling

**DOI:** 10.3390/ijms27031156

**Published:** 2026-01-23

**Authors:** Danielle Ferreira, Tayanne Sassaro, Anael Viana Pinto Alberto, Marília de Melo, Audrien Alves Andrade, Beatriz Iandra Ferreira, Otacílio C. Moreira, Daniel Moreira, Thiago Parente, Bruna Bordim, Júlia de Abreu, Fabiana Rondão, Jorge Canedo, Carlos Gil Ferreira, Elen de Souza, Aline Moreira, Mariana Waghabi, Mariano Gustavo Zalis, Tatiana Tilli

**Affiliations:** 1 Laboratory of Applied Genomics and Bioinovations, Oswaldo Cruz Institute, Oswaldo Cruz Foundation, Rio de Janeiro 21040-360, RJ, Brazil; danielle.pinto@fiocruz.br (D.F.); aanael@gmail.com (A.V.P.A.); daniel.andrade@fiocruz.br (D.M.); thiago.parente@fiocruz.br (T.P.); smoreiraaline@gmail.com (A.M.); mariana@ioc.fiocruz.br (M.W.); 2 Invitrocue Brasil, São Paulo 04513-020, SP, Brazil; bfolladorb@gmail.com (B.B.); juliaabreumsc@gmail.com (J.d.A.); 3 Integrated Center for Translational Oncology Research (CIPOT), Center for Technological Development in Health, Oswaldo Cruz Foundation, Rio de Janeiro 21040-900, RJ, Brazil; fabiana.rondao@gmail.com (F.R.); jorgeacanedo@gmail.com (J.C.); carlosgil.ferreira@oncoclinicas.com (C.G.F.); mgzalis@hucff.ufrj.br (M.G.Z.); 4 Next Generation Sequencing Facility, Oswaldo Cruz Foundation, Rio de Janeiro 21040-360, RJ, Brazil; mafimelo@gmail.com (M.d.M.); audrien.a.andrade@gmail.com (A.A.A.); 5 Molecular Virology and Parasitology Laboratory, Oswaldo Cruz Institute, Oswaldo Cruz Foundation, Rio de Janeiro 21040-360, RJ, Brazil; beatriziandra@outlook.com (B.I.F.); otacilio@ioc.fiocruz.br (O.C.M.); emello@ioc.fiocruz.br (E.d.S.); 6 Molecular Analysis Facility, Oswaldo Cruz Foundation, Rio de Janeiro 21040-360, RJ, Brazil; 7 Bioinformatics Core Facility, Oswaldo Cruz Foundation, Rio de Janeiro 21040-900, RJ, Brazil; 8 Oncoclinicas&Co.-Medica Scientia Innovation Research (MEDSIR), Sao Paulo 01304-001, SP, Brazil; 9 Laboratory of Clinical and Experimental Pathophysiology, Oswaldo Cruz Institute, Oswaldo Cruz Foundation, Rio de Janeiro 21040-360, RJ, Brazil

**Keywords:** patient-derived organoids, precision oncology, morphological profiling, tumor–virus interactions, SARS-CoV-2 infection models, SARS-CoV-2 variants, lung cancer, breast cancer, colorectal cancer

## Abstract

Cancer patients are highly vulnerable to severe COVID-19, requiring models that capture tumor–virus interactions. We investigated tumor- and variant-specific effects of SARS-CoV-2 Gamma and Delta infections using patient-derived organoids (PDOs) from metastatic breast, lung, and colorectal cancers. Viral infection was quantified by Real-Time Quantitative Polymerase Chain Reaction (RT-qPCR) 24 h post-infection, and morphological changes and immune mediators were profiled. Genomic analysis using whole-exome sequencing was performed to identify contributing host-related gene alterations. The Delta variant produced consistently higher viral loads in lung and breast PDOs, while colorectal PDOs showed variable susceptibility. Infection led to reduced area and perimeter and increased circularity across all tumor types. Immune profiling revealed distinct responses: Gamma decreased Interferon alpha (IFNα) in lung PDOs and increased E-selectin in colorectal PDOs. Delta broadly reduced inflammatory mediators in lung [10 kDa interferon gamma-induced protein (IP-10) and Intercellular adhesion molecule 1 (ICAM-1)] and breast [Interleukin-6 (IL-6), Interleukin-13 (IL-13), and Interleukin-17A (IL-17A)] PDOs, while increasing Macrophage inflammatory protein 1-beta (MIP-1β) in colorectal PDOs. Host gene variants involved in trafficking (*FYCO1* and *RAB7A*) and immune signaling (*FOXA2*, *SFTPD*, *STAT3*, and *TET2*) were associated with differential infection profiles. These findings show that SARS-CoV-2 induces variant- and tumor-specific morphological and immunological changes in cancer PDOs, highlighting the potential of this model to unravel host–virus interactions and identify genetic factors that shape infection outcomes in cancer.

## 1. Introduction

COVID-19 presents a wide spectrum of clinical manifestations, ranging from asymptomatic cases to severe respiratory failure and multi-organ dysfunction [1]. Cancer patients, who often have compromised immune system, are particularly vulnerable to severe outcomes from COVID-19. Studies have shown that individuals with cancer have a higher risk of hospitalization, severe complications, and mortality following SARS-CoV-2 infection compared to the general population [2,3,4,5]. This increased susceptibility is primarily attributed to the immunosuppressive effects of cancer itself, as well as the impact of treatments such as chemotherapy, radiation, and immunotherapy [6]. However, despite extensive clinical characterization, the interaction between SARS-CoV-2 and the tumor microenvironment remains poorly understood. Importantly, while the systemic effects of COVID-19 worsen clinical outcomes in cancer patients, the impact of the infection on tumor cells themselves may not follow the same detrimental pattern. In fact, emerging evidence suggests that viral infection could exert cytotoxic or modulatory effects on cancer cells, making the tumor context a unique environment for studying virus–tumor interactions [7,8].

A hallmark of immune response in COVID-19 is hyperinflammation, often referred to as a cytokine storm, which is characterized by excessive and uncontrolled production of pro-inflammatory cytokines [9,10]. Given that tumor cells secrete various immune-regulating cytokines [11] and that SARS-CoV-2 infection may further disrupt cytokine balance in cancer patients, the tumor microenvironment may be altered, potentially influencing therapeutic strategies. Additionally, there is ongoing debate about whether viral infection can trigger anti-tumor immune responses or instead promote tumor progression, making this an area of significant translational importance.

One of the defining features of SARS-CoV-2 variants is their differential infectivity and impact on public health. The Gamma variant (P.1) caused severe outbreaks due to its increased transmissibility compared to the original strain [12]. The Delta variant, which subsequently became the dominant strain in multiple regions, exhibited even higher transmissibility than the Gamma variant. Although their clinical impact in the general population is documented, the specific effects of these variants on cancer cells and tumor microenvironments remain poorly understood.

Given the dual burden of cancer and COVID-19, it is essential to investigate how SARS-CoV-2 infection affects tumor cells and the tumor microenvironment. This study aims to explore the effects of Gamma and Delta variant infections on patient-derived organoids from breast, lung, and colorectal cancers. By analyzing viral load, morphological changes, cytokine release, and whole-exome sequencing data, we seek to investigate how SARS-CoV-2 influences tumor biology and immune responses. Our results show that SARS-CoV-2 infection reduces patient-derived organoid (PDO) size in a variant- and tumor type-dependent manner and modulates cytokines and immune mediators, with exploratory exome data suggesting potential gene-level contributors.

## 2. Results

### 2.1. Biological Samples and Patient Characteristics

Organoids were successfully generated from tumor biopsies of 17 patients diagnosed with metastatic cancers, including breast, lung, and colorectal cancers. Samples were derived from both primary and metastatic sites. A total of eight patients (47%) were diagnosed with lung cancer, followed by four (24%) with breast cancer and five (29%) with colorectal cancer. The study population included 10 female and 7 male patients (Table 1).

### 2.2. Establishment of Patient-Derived Organoids (PDOs)

**Figure 1:**
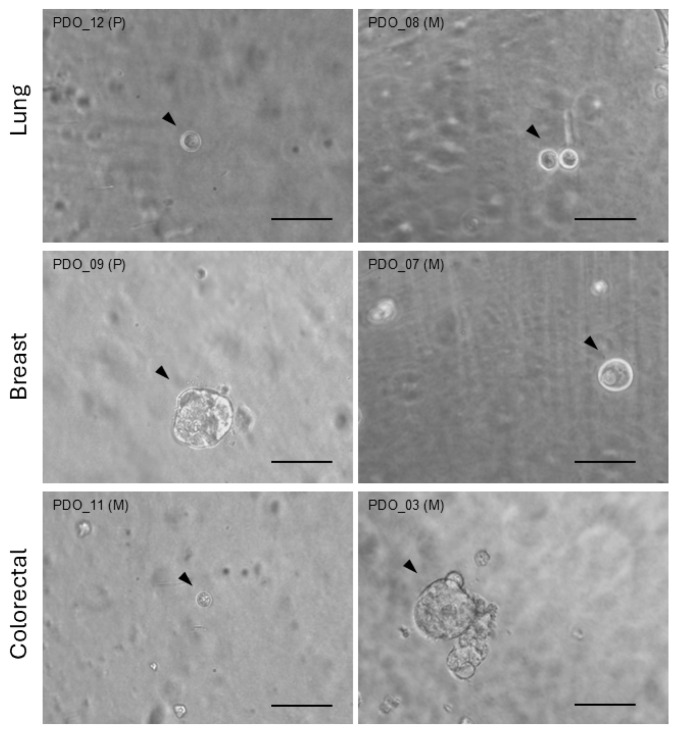
Organoid images. Bright-field images of representative organoids (arrow heads) generated from distinct primary (P) or metastatic (M) biopsies from patients with lung, breast, or colorectal cancer. Scale bar = 100 µm.

From patient biopsy material, organoids were generated from a uniform pool of cells. Due to their self-organizing capacity, organoids within a given group or condition could exhibit variability in quantity, size, and shape. Figure 1 displays examples of organoids from different patients within each cancer type group.

### 2.3. Genomic Characterization of PDOs via Whole-Exome Sequencing

**Figure 2:**
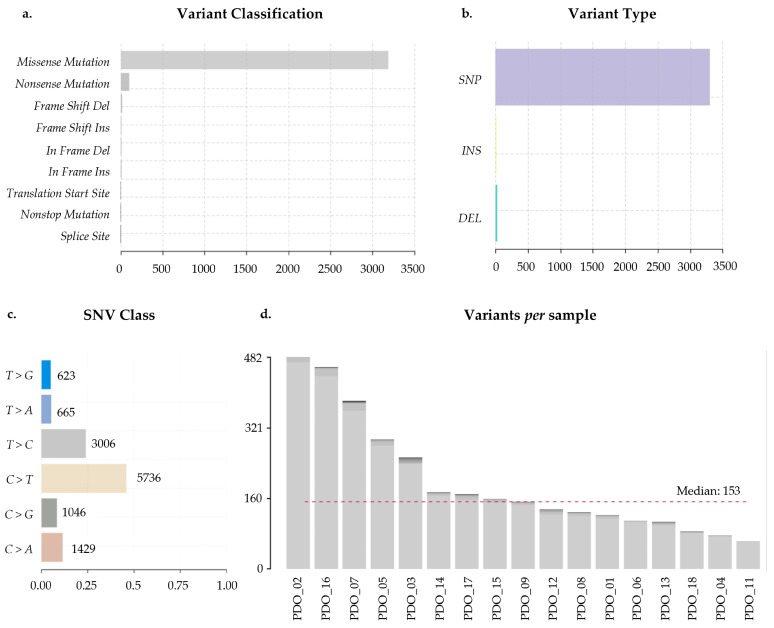
Whole-exome sequencing (WES) variant analysis. (**a**) Distribution of variant classifications detected across all samples, including missense, nonsense, frameshift, in-frame indels, and splice-site variants. (**b**) Variant types identified, categorized as single nucleotide polymorphisms (SNPs), insertions (INS), and deletions (DEL). (**c**) Single nucleotide change (SNC) spectrum, showing the frequency of each substitution class. (**d**) Number of variants detected per sample. The dashed red line indicates the cohort median. A total of 17 biological samples (lung = 8, breast = 4, and colorectal = 5) were included in the analysis. Variants were called using GATK Mutect2 and Strelka, annotated with Variant Effect Predictor (VEP) and visualized using maftools. Only high-confidence variants passing all filtering criteria were used.

Whole-exome sequencing (WES) was performed on all samples, and data was processed through an initial pipeline to obtain the variant call format (VCF) file. Analysis of the VCF revealed that 95% of the nonsynonymous variants were classified as missense mutations (Figure 2a). Regarding variant type, single nucleotide polymorphisms (SNPs) were the most prevalent (99%) (Figure 2b). Among single nucleotide variations (SNVs), the most common nucleotide substitution was C>T, followed by T>C, accounting for 46% and 24% of all nucleotide changes, respectively (Figure 2c).

PDO_02 exhibited the highest number of nonsynonymous variants (482), followed by PDO_16 (460) and PDO_07 (383). Conversely, PDO_11, PDO_04, and PDO_18 presented the lowest number of variants (62, 76, and 85, respectively). The median of nonsynonymous variants detected across all samples was 153 (Figure 2d).

### 2.4. SARS-CoV-2 Infection and Viral Load Quantification

Organoid cultures were infected with SARS-CoV-2 Gamma or Delta variants at a multiplicity of infection (MOI) of 0.01 for 24 h. DNA was then extracted from each sample, and the presence of the N2 gene target was assessed to confirm successful infection. Viral load was detected for both variants, indicating active viral replication within the organoids.

**Figure 3:**
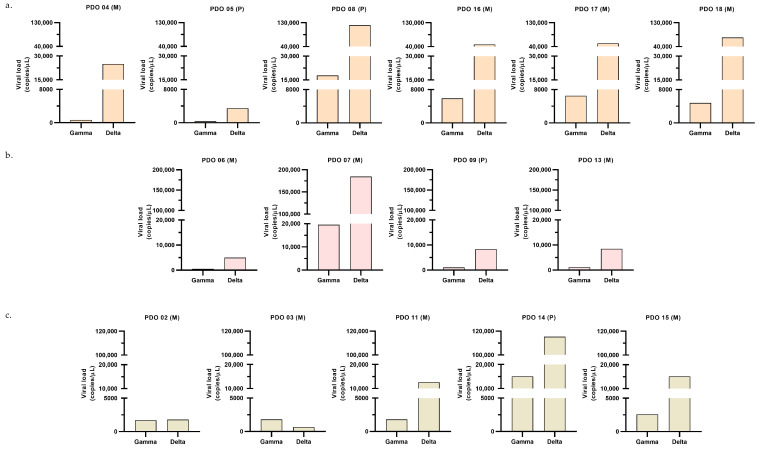
Post-infection viral load. Viral load quantified by RT-qPCR 24 h after infection with Gamma or Delta variants, expressed as viral copies/µL. Each graph shows data from a distinct PDO [sample ID, biopsy site (P: primary; M: metastatic)]; for each PDO, one experiment was performed, and each bar corresponds to the mean of two technical replicates from the culture supernatant. Panels correspond to (**a**) lung (orange graphs); (**b**) breast (pink graphs); (**c**) colorectal (beige graphs).

**Figure 4:**
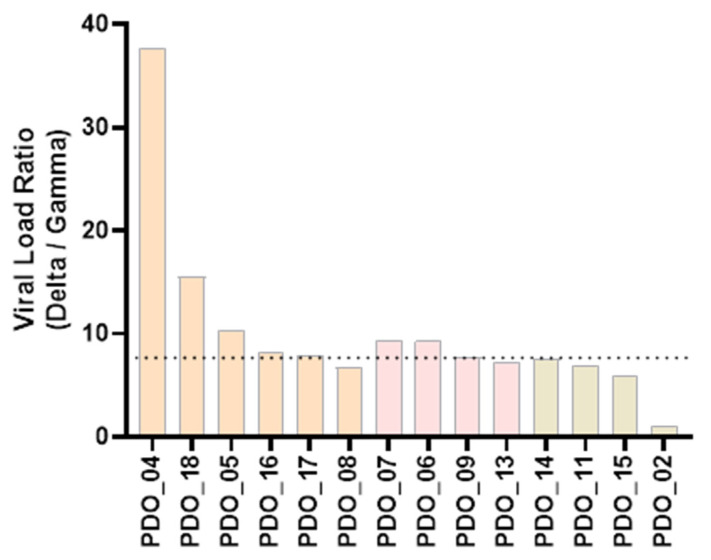
Delta-to-Gamma viral load ratio. Each bar represents the ratio of one patient. Orange bars indicate samples from lung cancer; pink bars, from breast cancer; and beige bars, from colorectal cancer. Median value is shown as a dotted line.

Susceptibility of PDOs from different cancer types to Gamma and Delta was evaluated based on viral load, with higher values indicating greater susceptibility. In lung and breast cancer organoids, the Delta variant consistently exhibited higher viral loads than Gamma (Figure 3a,b). In contrast, colorectal cancer organoids displayed a heterogeneous pattern: organoids from three patients showed greater susceptibility to Delta variant, resembling the lung and breast profiles; on the other hand, organoids from one patient (PDO_03) were more susceptible to Gamma than Delta; and organoids from another patient (PDO_02) exhibited comparable viral loads for both variants (Figure 3c). Notably, most of the samples that exhibited greater susceptibility to Delta variant, compared to Gamma, displayed a Delta-to-Gamma viral load ratio around 7 (median = 7.80), as indicated in Figure 4. These results indicate that PDOs from all cancer types are susceptible to infection by both variants. Having established infection susceptibility, we then assessed how this exposure translated into variant- and tumor-type-specific biological effects.

### 2.5. Impact of SARS-CoV-2 Infection on Organoid Morphology

**Figure 5:**
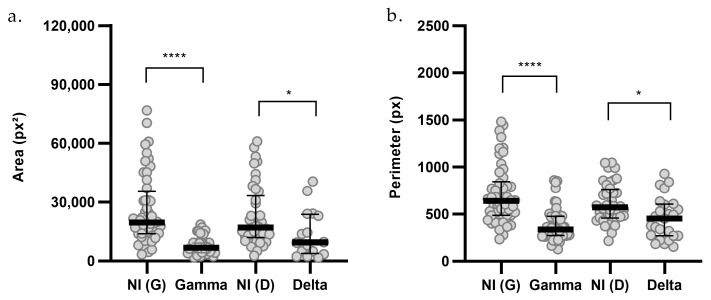
Morphometric analysis of (**a**) area and (**b**) perimeter of organoids infected with SARS-CoV-2 variants. Non-infected Gamma controls [NI(G), *n* = 48], Gamma-infected organoids (G, *n* = 37), non-infected Delta controls [NI(D), n = 41], and Delta-infected organoids (D, *n* = 16) are shown. *n* indicates the total number of individually measured organoids per group. Statistical analyses were performed using Kruskal–Wallis test, followed by Dunn’s multiple comparisons test. Statistical significance: * *p* < 0.05; **** *p* < 0.0001.

Morphological analyses of organoids were performed using 2D bright-field images. A total of 184 single organoids were analyzed across groups infected with the Gamma and Delta variants and their respective controls, for samples derived from lung (PDO_04, PDO_08, PDO_12), breast (PDO_07, PDO_09), and colorectal (PDO_03, PDO_11) cancer patients. The analysis revealed a reduction in organoid area following infection with both Gamma and Delta variants (Figure 5a). Gamma infection led to a 65% decrease in median organoid area [G = 6768; NI(G) = 19,480; *p* < 0.0001], while Delta infection reduced the area by 44% [D = 9434; NI(D) = 16,968; *p* = 0.0351].

Additionally, perimeter analyses indicated that infection with both the Gamma and Delta variants resulted in reduced organoid size (Figure 5b). Gamma infection led to a 48% decrease in organoid perimeter [G = 335.7; NI(G) = 639.1; *p* < 0.0001], and Delta infection reduced the perimeter by 21% [D = 451.9; NI(D) = 570.4; *p* = 0.0143].

**Figure 6:**
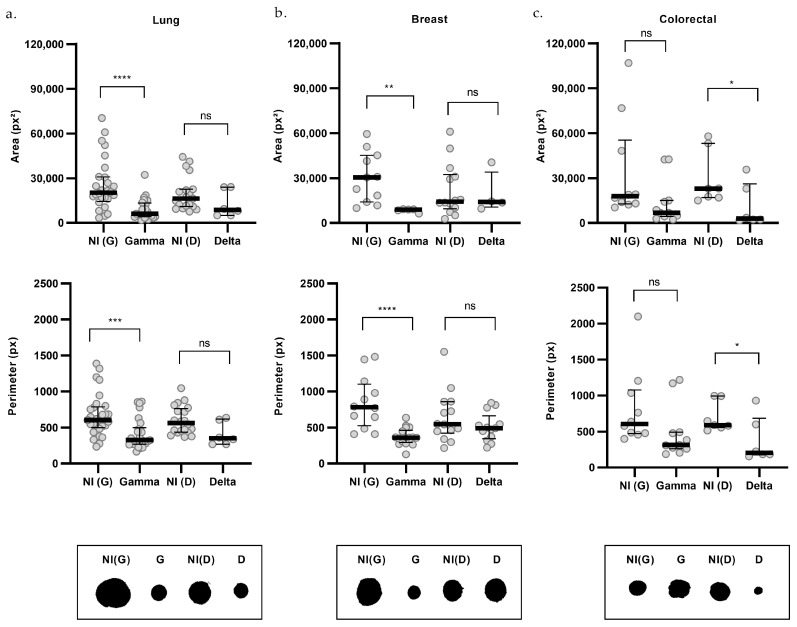
Morphometric analysis by cancer type of organoids infected with SARS-CoV-2 variants. Upper graphs show organoid area, middle graphs show perimeter, and lower panels present representative morphologies for lung (**a**), breast (**b**), and colorectal (**c**) organoids. Non-infected controls (NI) are shown in gray, Gamma-infected organoids (G) in blue, and Delta-infected organoids (D) in purple. For area: lung NI(G) (*n* = 28), G (*n* = 24), NI(D) (*n* = 19), D (*n* = 6); breast NI(G) (*n* = 11), G (*n* = 5), NI(D) (*n* = 14), D (*n* = 4); and colorectal NI(G) (*n* = 10), G (*n* = 11), NI(D) (*n* = 7), D (*n* = 6). For perimeter: lung NI(G) (*n* = 29), G (*n* = 26), NI(D) (*n* = 20), D (*n* = 6); breast NI(G) (*n* = 12), G (*n* = 18), NI(D) (*n* = 14), D (*n* = 13); and colorectal NI(G) (*n* = 10), G (*n* = 11), NI(D) (*n* = 7), D (*n* = 6). *n* indicates the number of individually measured organoids per group. Statistical analyses were performed using Kruskal–Wallis test, followed by Dunn’s multiple comparisons test. Statistical significance: ns = not significant; * *p* < 0.05; ** *p* < 0.01; *** *p* < 0.001, **** *p* < 0.0001.

The impact of infection on organoid size was then assessed across each cancer type (Figure 6). Gamma infection significantly reduced both area and perimeter in lung (Figure 6a) and breast cancer organoids (Figure 6b). The reduction in area was 70% in lung [G = 6115; NI(G) = 20,223; *p* < 0.0001] and breast organoids [G = 8998; NI(G) = 30,447; *p* = 0.0024], while the perimeter decreased by 46% and 54% in lung [G = 326.1; NI(G) = 603.7; *p* = 0.0004] and breast (G = 359.1; NI(G) = 779.6; *p* < 0.0001] organoids, respectively. However, Gamma infection did not produce significant changes in area [G = 6821; NI(G) = 17,832] or perimeter (G = 313.9; NI(G) = 606.4] in colorectal cancer organoids (Figure 6c).

Delta infection, on the other hand, led to a reduction in both area [D = 2938; NI(D) = 23,002; *p* = 0.0222] and perimeter [D = 204.4; NI(D) = 590.2; *p* = 0.0415], specifically in colorectal cancer organoids, by 87% and 65%, respectively (Figure 6c). However, these parameters were not significantly altered in lung [area: D = 8709; NI(D) = 16,326; perimeter: D = 350.4; NI(D) = 563.8] or breast cancer organoids [area: D = 14,079; NI(D) = 14,299; perimeter: D = 491.5; NI(D) = 546.1; Figure 6a,b].

**Figure 7:**
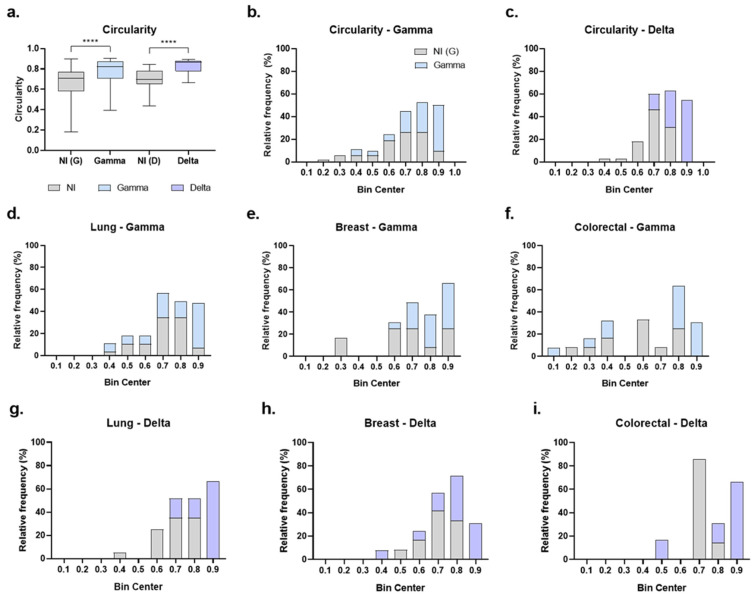
Circularity of cancer organoids after SARS-CoV-2 infection. (**a**) Circularity of organoids from all cancer types combined; non-infected Gamma controls [NI(G), *n* = 53], Gamma-infected (Gamma, *n* = 54), non-infected Delta controls [NI(D), *n* = 39], and Delta-infected (Delta, *n* = 22). Data shown as boxplots with interquartile range; n indicates the total number of individually measured organoids per group. (**b**,**c**) Circularity distributions (bin width = 0.1) comparing NI(G) vs. Gamma (**b**) and NI(D) vs. Delta (**c**). (**d**–**f**) Gamma: lung [NI(G), *n* = 29; Gamma, *n* = 29], breast [NI(G), *n* = 12; Gamma, *n* = 21], colorectal [NI(G), *n* = 12; Gamma, *n* = 13]. (**g**–**i**), Delta: lung [NI(D), *n* = 20; Delta, *n* = 6], breast [NI(D), *n* = 14; Delta, *n* = 13], colorectal [NI(D), *n* = 7; Delta, *n* = 7]. Gray bars/boxes represent non-infected controls, blue represent Gamma-infected, and purple represent Delta-infected groups. *n* indicates the number of individually measured organoids per group. Statistical analyses were performed using Kruskal–Wallis test, followed by Dunn’s multiple comparisons test. Statistical significance: **** *p* < 0.0001.

Another morphological aspect assessed to identify potential changes induced by SARS-CoV-2 Gamma and Delta variants in tumor organoids was circularity, with values closer to 1 indicating a more circular shape and less elongated form. The data indicated that organoids became more circular following infection with both the Gamma [G = 0.824; NI(G) = 0.709; *p* < 0.0001] and Delta [D = 0.867; NI(D) = 0.699; *p* < 0.0001] variants, compared to their respective non-infected controls (Figure 7a). Importantly, circularity was not statistically different between non-infected control groups (*p* > 0.05).

Circularity values of organoids were then organized into histograms with a bin range of 0.1 to better visualize the effect (Figure 7b–i). In the non-infected groups, organoid circularity predominantly ranged between 0.6 and 0.9 in the Gamma control group (Figure 7b) and between 0.6 and 0.8 in the Delta control group (Figure 7c), corresponding to 92% and 83% of organoids, respectively. For infected groups, we observed a shift in distribution toward a more circular form. Among Gamma-infected organoids, 94% fell within 0.7 to 0.9 bins (Figure 7b). For the Delta-infected group, 85% were distributed within the 0.7 to 0.9 bins (Figure 7c). Notably, the most circular organoid (0.9 bin) were predominantly infected. Compared to their respective controls, 70% of organoids in the 0.9 bin were infected with Gamma (Figure 7b), and 100% were infected with Delta (Figure 7c).

Analysis of circularity by cancer type also revealed a shift toward a more circular shape. The most circular bin (0.9) was predominantly composed of infected organoids across all cancer types (Figure 7d–i). Specifically, for Gamma infection, 85% of organoids in the 0.9 bin were from the infected group in lung cancer (Figure 7d), 70% in breast cancer (Figure 7e), and 100% in colorectal cancer (Figure 7f). For Delta infection, 100% of organoids in the 0.9 bin were infected in all lung, breast, and colorectal cancers (Figure 7g–i).

These findings indicate that SARS-CoV-2 infection induces consistent morphological remodeling, but the magnitude and features of this response are shaped by both the viral variant and the tumor type. To determine whether these structural changes were accompanied by alterations in inflammatory signaling, we next examined the profile of immunological mediators released upon infection.

### 2.6. Immunological Response of PDOs to SARS-CoV-2 Variants

SARS-CoV-2 infection is known to induce dysregulation in immunological and inflammatory mediators. Therefore, the levels of multiple soluble immune mediators were measured in the supernatant of organoid cultures 24 h post-infection with the Gamma and Delta variants, including cytokines [Granulocyte-macrophage colony-stimulating factor (GM-CSF), Interferon alpha (IFN-α), Interferon gamma (IFN-γ), Interleukin-1 alpha (IL-1α), Interleukin-1 beta (IL-1β), Interleukin-4 (IL-4), Interleukin-6 (IL-6), Interleukin-8 (IL-8), Interleukin-10 (IL-10), Interleukin-12p70 (IL-12p70), Interleukin-13 (IL-13), Interleukin-17A (IL-17A), and Tumor necrosis factor (TNF-α)], chemokines [10 kDa interferon gamma-induced protein (IP-10), Monocyte chemotactic protein 1 (MCP-1), Macrophage inflammatory protein 1-alpha (MIP-1α), and Macrophage inflammatory protein 1-beta (MIP-1β)], and molecules related to cell adhesion and inflammatory response [Intercellular adhesion molecule 1 (ICAM-1), E-selectin, and P-selectin].

**Figure 8:**
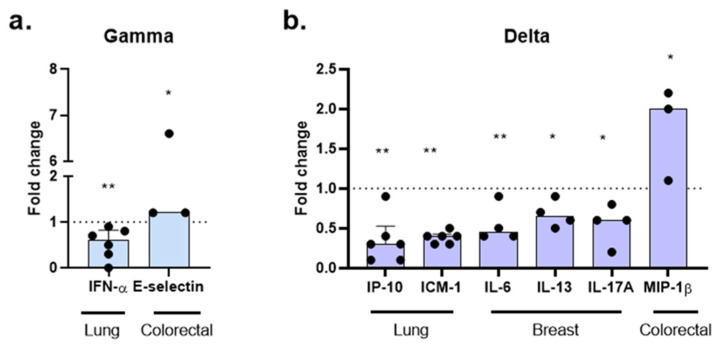
Modulation of immune mediators by SARS-CoV-2 variants in cancer organoids. Molecules were assessed 24 h post-infection with Gamma (**a**) and Delta (**b**) variants. Data are presented as fold change (non-infected controls normalized to 1, shown as a dotted line). Each point corresponds to the supernatant from the organoid culture of an individual patient: lung (*n* = 6), breast (*n* = 4), and colorectal (*n* = 3). Statistical analyses were performed using Kruskal–Wallis test, followed by Dunn’s multiple comparisons test. Statistical significance: * *p* < 0.05; ** *p* < 0.01.

Among the 20 molecules analyzed, tumor type-specific analyses revealed that each variant induced distinct patterns in the release of these mediators. In lung cancer organoids, infection with the Gamma variant resulted in a 40% reduction in IFN-α secretion (fold change [FC] = 0.6; *p* = 0.008). In contrast, in colorectal organoids, Gamma infection led to a 20% increase in E-selectin levels (FC = 1.2; *p* = 0.0436) (Figure 8a).

The Delta variant also produced tumor type-dependent effects. In lung cancer organoids, Delta infection induced significant reductions in IP-10 and ICAM-1 levels, with 70% (FC = 0.3; *p* = 0.0027) and 60% (FC = 0.4; *p* = 0.0072) decreases, respectively. In breast cancer organoids, Delta infection resulted in decreased secretion of IL-6 (FC = 0.45; *p* = 0.0099), IL-13 (FC = 0.65; *p* = 0.0483), and IL-17A (FC = 0.6; *p* = 0.0282). In contrast, in colorectal cancer organoids, Delta infection promoted a twofold increase in MIP-1β levels (FC = 2.0; *p* = 0.0365), compared to non-infected controls. No significant differences were observed for the release of other molecules in Gamma- or Delta-infected groups.

These findings indicate that infection with different SARS-CoV-2 variants modulates the release of specific mediators of the immune and inflammatory response in a tumor-type-dependent manner, highlighting the role of the tumor microenvironment in the response to viral infection.

To investigate whether phenotypic and inflammatory effects might be linked to underlying genomic differences across PDOs, we next examined whether somatic mutations in infection-related host genes could contribute to the observed responses.

### 2.7. Genetic Variants in SARS-CoV-2 Host-Related Genes and Their Association with Viral Load in PDOs

Given that exonic mutations can alter protein structure and function, we focused our analysis on nonsynonymous variants within host genes implicated in viral entry, replication, and host immune response. A curated list of the genes included in this analysis is provided in Appendix A (Table A1).

Across the dataset, and excluding synonymous variants, we identified 13 missense variants, 2 variants located in the 3′ untranslated region (3′ UTR), 1 upstream gene variant, and 1 intron variant (Table 2). To explore whether these alterations could be associated with the differences observed in viral load, we stratified our analysis by cancer type.

Among lung cancer samples, PDO_05 exhibited the lowest viral load. This sample harbored a unique missense variant in the gene *FYCO1*, resulting in an amino acid substitution from alanine to serine.

Conversely, in the breast cancer cohort, PDO_07 showed a markedly higher viral load compared to the other PDOs. Two missense variants were exclusively detected in this PDO_07: *FOXA2* (D→N amino acid change) and *SFTPD* (E→K amino acid change). Other breast PDOs lacked these variants and displayed substantially lower viral loads.

In colorectal cancer PDOs, PDO_02 and PDO_03 exhibited lower viral loads than the other samples and both deviate from the general pattern of higher Delta replication. Notably, PDO_02 displayed similar viral loads for both variants, whereas PDO_03 had higher levels following Gamma infection. Exclusive missense variants in *STAT3* (P→T amino acid change) and *TET2* (F→L amino acid change) were found in PDO_02. No exclusive coding mutations were found in PDO_03; however, both PDO_03 and PDO_05 shared a 3’UTR variant in *RAB7A*. Despite this shared variant, these two PDOs did not present common features in viral load profiles that would support a shared phenotype.

Altogether, the combined evidence of productive infection, organoid shrinkage, and context-dependent inflammatory response points to a potential oncolytic effect exerted by SARS-CoV-2 in cancer-derived models. Although the limited sample size is insufficient to support definitive conclusions, this integrated phenotypic and genotypic approach highlights the influence of tumor-intrinsic factors on viral susceptibility and immune signaling. This study opens new perspectives for translational studies at the intersection of virology and oncology, particularly considering the distinct biological effects observed between the Gamma and Delta SARS-CoV-2 variants in cancer-derived models.

## 3. Discussion

Organoids have become valuable tools in cancer research by closely replicating the 3D structure and biology of tumors, including genetic, histological, and microenvironmental features [13,14,15]. Their preservation of cellular heterogeneity, signaling pathways, and tissue architecture makes them suitable models for studying tumor interactions with viruses [16,17]. In this study, we examined how SARS-CoV-2 Gamma and Delta variants affect patient-derived organoids from lung, breast, and colorectal cancers.

We confirmed that tumor cells are permissive to SARS-CoV-2 infection. We confirmed that tumor cells are permissive to SARS-CoV-2 infection. Most of the analyzed PDOs exhibited higher viral loads following infection with the Delta variant compared to Gamma, with Delta-to-Gamma ratios typically around 7-fold. This trend aligns with previous reports describing increased replication and viral loads for the Delta variant relative to earlier SARS-CoV-2 variants [18,19]. In the present study, we described this effect in tumor cells using patient-derived organoids.

We observed differential susceptibility among samples, which could lead to significant changes in the tumor microenvironment and imply that SARS-CoV-2 variants may affect cancer patients differently. Viral entry could not only influence inflammatory response but also affect disease progression. Samples that exhibited viral loads that deviated markedly from the typical pattern seen in their cancer group were particularly intriguing. To explore this, we analyzed whole-exome sequencing data and identified mutations potentially affecting viral entry and immune response.

For this purpose, we highlight genes that may be related to the deviated effects observed within each cancer type group. In lung cancer organoids, *FYCO1* has been implicated in cellular responses to viral infections through its role in autophagosome transport along microtubules. Integrative analyses identified *FYCO1* as an important host gene associated with SARS-CoV-2 infection [20]. In our study, a lung sample (PDO_05) carrying a missense mutation in *FYCO1* showed a significantly reduced viral load following infection. Although further mechanistic studies are required, it is plausible that this mutation impairs *FYCO1* function, limiting the trafficking of membranous compartments required for efficient viral replication.

In breast cancer organoids, PDO_07 exhibited the highest viral load among the samples from this tumor type, potentially linked to its unique genetic profile. This PDO harbored missense variants in *FOXA2* and *SFTPD*, genes involved in regulating viral entry and host defense. *FOXA2* encodes a transcription factor that preserves epithelial barrier integrity and regulates innate immune genes, including surfactant proteins. A mutation in *FOXA2* may impair these protective functions, increasing cellular permissiveness to infection. *SFTPD* encodes surfactant protein D, which binds the SARS-CoV-2 Spike protein and inhibits its interaction with ACE2 [21]. A mutation in this gene may disrupt viral neutralization. Together, these variants could contribute to the enhanced viral replication observed in PDO_07, suggesting a potential genetic predisposition affecting both susceptibility and host defense.

In colorectal cancer, PDO_02 carries missense mutations in *STAT3* and *TET2*, key regulators of innate immunity and inflammation. STAT3 mediates inflammatory signaling, particularly downstream of IL-6, and its dysregulation has been linked to impaired antiviral responses and cytokine storm in COVID-19 [22]. *TET2* regulates immune gene expression epigenetically, and loss-of-function mutations have been associated with enhanced pro-inflammatory cytokine production and greater vulnerability to severe viral infections [23,24]. Unlike other PDOs where Delta showed higher replication, PDO_02 exhibited similar viral loads for the Delta and Gamma variants. Cytokine quantification was not feasible in this sample, limiting the ability to directly correlate genotype and inflammatory response.

PDO_03, another colorectal sample, displayed an atypical infection profile, with markedly lower viral load after Delta infection—even below levels induced by Gamma. This PDO harbors a 3’ UTR variant in *RAB7A*, a gene involved in endosomal trafficking and viral processing. Loss of *RAB7A* function has been associated with reduced viral entry [25]. Although no coding mutations were detected in infection-related genes in this sample, the 3’ UTR variant may affect post-transcriptional regulation, such as mRNA stability or microRNA binding, potentially altering *RAB7A* expression. Notably, the same variant was found in PDO_05 (lung), which showed elevated Delta viral load and carries an additional *FYCO1* mutation. *FYCO1* and *RAB7A* are functionally connected within the same vesicular trafficking pathway. While PDO_05 exhibited higher Delta than Gamma viral load (though still lower than in other lung PDOs), PDO_03, with only the *RAB7A* variant, showed the opposite trend. These contrasting viral phenotypes suggest that tumor type-specific factors or other genetic alterations may modulate viral replication and cellular permissiveness.

Taken together, these results illustrate the complex interplay between host genetic variation and SARS-CoV-2 infection dynamics across different tumor types. Mutations in genes related to innate immunity, viral trafficking, and epithelial integrity appear to influence viral load and variant-specific susceptibility in a context-dependent manner. While some variants may enhance infectivity, others could confer resistance to viral replication, underscoring heterogeneity in host–virus interactions in cancer. These insights contribute to a better understanding of COVID-19 pathogenesis in oncology and highlight potential genetic factors relevant for personalized risk assessment and therapeutic development. Future functional studies and larger cohorts will be essential to clarify the mechanisms underlying these observations.

The morphology of PDOs provides valuable insights into the functional and structural state of tumor cells, as assessed by parameters such as area, perimeter, and circularity. Morphological analysis revealed a decrease in both area and perimeter in PDOs infected with both Delta and Gamma variants, suggesting a reduction in cell number or overall tissue volume, which may indicate direct viral cytotoxicity, inhibited cell proliferation, or tissue remodeling. This aligns with studies showing that viral infections can disrupt cellular homeostasis and induce apoptosis or necrosis in various cell types [26,27]. However, when analyzed by cancer type, distinct variant-specific responses emerged, indicating tissue-specific interactions between SARS-CoV-2 and tumor cells. Although infection with the Gamma variant resulted in lower viral loads, it induced a more pronounced reduction in lung and breast PDO area and perimeter when compared to the Delta variant, which presented no significant effect. In contrast, the Delta variant decreased area and perimeter in colorectal cancer PDOs, suggesting distinct morphological effects associated with each variant with specific effects dependent on tumor type.

Interestingly, we also observed a significative overall increase in PDO circularity following infection with both variants. Interestingly, the most circular organoids (0.9 bin) were predominantly found among infected groups. A stronger association was evident with the Delta variant, where all PDOs falling into this highest circularity bin were infected. These findings suggest that infection with Gamma and Delta, particularly Delta, may induce a specific cellular reorganization that favors a more spherical morphology. The effect of reducing PDO size and increasing circularity suggests that SARS-CoV-2 infection could have an anti-tumor effect, acting as an oncolytic virus. This concept is further supported by observations in cell line studies and simulations, which correlate branched and elongated morphologies with a more aggressive and invasive cancer phenotype [28,29], although it is not a consensus. Moreover, a study using prostate cancer organoids demonstrated the reversal of an irregular/slithering morphology following anti-cancer treatment [29]. This highlights the potential of morphological analysis as a valuable indicator of cellular aggressiveness and treatment response or tumor progression. Therefore, in this context, an increase in circularity, as observed in our results, could indicate reduced tumor aggressiveness.

Indeed, studies in the literature suggest that SARS-CoV-2 may have the potential to act as an oncolytic virus, reducing tumor size. The concept of oncolytic viruses, agents that selectively destroy cancer cells, has gained increasing attention in cancer therapy. While SARS-CoV-2’s impact on cancer patients presents a complex and often contradictory picture, with some studies reporting worsened outcomes [30,31], a growing body of evidence, including case reports, mathematical modeling and experimental studies, suggests potential anti-tumor effects across hematological and solid malignancies, including breast, lung, and colorectal cancers [28,32,33].

The mechanisms underlying these effects remain largely elusive, but several hypotheses have emerged, such as that SARS-CoV-2 infection renders tumor cells more vulnerable to immune-mediated destruction [32]; that virus-induced cytokine dysregulation mediates oncolysis [34,35]; and that decreased expression of genes associated with cancer proliferation and metastasis following SARS-CoV-2 contributes to tumor regression [8]. As highlighted by previous reports [8], acute viral replication can induce direct lytic cell death or stimulate antiviral immune responses, thereby modulating the tumor immune microenvironment.

While many reported remission effects are systemic, our in vitro study, using tumor organoids, suggests that SARS-CoV-2 may exert direct tumor-modulating effects, leading to measurable phenotypic changes. These effects may result from virus-induced modulation of cytokines and/or other tumor-intrinsic mechanisms. Further investigation is needed to elucidate these pathways and uncover their potential implications for cancer–virus interactions.

Our cytokine analysis revealed significant effects in the release of specific cytokines following infection with the Gamma and Delta variants in a cancer type-specific context.

In lung cancer PDOs, infection with the Delta variant led to a significant reduction in IP-10 and ICAM-1 levels. These molecules play key roles in immune cell signaling and tumor-related processes. ICAM-1, involved in cell adhesion and interactions between immune and tumor cells, plays a role in tumor metastasis by facilitating interactions with endothelial cells and modulating immune responses. IP-10 is a chemokine that coordinates chemotaxis of immune cells, especially T lymphocytes, to inflammatory and tumor sites, and traditionally exhibits potential anti-tumor effects. Conversely, IP-10 has also been shown to promote tumor progression in several studies. Elevated expression of IP-10 and its receptor, CXCR3, has been associated with increased metastatic potential and poor prognosis [36,37,38]. IP-10 has been reported to promote cell proliferation and survival, while CXCR3 ligation enhances tumor invasiveness through increased motility [38]. Notably, Zhu et al. [37] demonstrated that CXCR3 blockade impaired tumor dissemination and concurrently enhanced host anti-tumor immune responses.

Therefore, the observed reduction in IP-10 and ICAM-1 following Delta variant infection suggests an effect favoring an anti-tumor response. This reduction, particularly of IP-10, may disrupt the pro-tumorigenic signaling pathways mediated by CXCR3, potentially limiting tumor cell migration, proliferation, and immunosuppression. Consequently, the observed reduction in cytokine release following Delta variant infection reinforce the oncolytic potential of this variant by highlighting a unique immunomodulatory mechanism.

Interestingly, a distinct cytokine modulation pattern was observed with the Gamma variant, which led to a significant decrease in IFN-α levels. IFN-α is a type I interferon with a central role in antiviral immunity, but its function in cancer remains controversial, as it can exert both anti- and pro-tumorigenic effects depending on the cellular and molecular context. The reduction in IFN-α observed exclusively with Gamma suggests that this variant may interact specifically with the type I interferon pathway in lung tumor cells. This finding aligns with prior observations by Chen et al. [39], who demonstrated that SARS-CoV-2 infection can suppress components of the JAK-STAT signaling cascade, thereby inhibiting interferon responses across several cell types, including A549 lung tumor cells. Notably, their study used the original strain (2019-nCoV/USA-WA1/2020), indicating that Gamma may share similar immunosuppressive features with early variants, whereas Delta appears to have lost this capacity.

Morphological analysis revealed that Gamma infection significantly reduced the area and perimeter of lung organoids. Although circularity was not significantly altered, there was a trend toward increased circularity. These findings may indicate that Gamma affects the structural organization of the organoids without inducing major disruptions in their overall shape. Unlike Delta, which showed concurrent effects on both morphology and cytokine release, Gamma’s effects may reflect different underlying mechanisms, possibly involving modulation of interferon pathways or other regulatory processes within the tumor cells.

In breast cancer PDOs infected with SARS-CoV-2, our study revealed a distinct cytokine response characterized by a significant decrease in IL-6, IL-13, and IL-17A release at 24 h post-infection with the Delta variant. These cytokine reductions are particularly intriguing given the well- known pro-tumorigenic roles of IL-6 and IL-17A in breast cancer, as well as the complex and potentially tumor-promoting effect of IL-13.

IL-6 has been strongly implicated in promoting breast cancer cell proliferation, survival, and metastasis, often correlating with poorer prognosis and advanced disease [40,41]. Similarly, IL-17A plays a key role in breast cancer progression, driving inflammation and promoting tumor growth and metastasis [42,43]. High IL-17A levels correlate with poorer prognosis and suggest its potential as a breast cancer marker and therapeutic target [44,45]. IL-13, known for its role in Th2-mediated immune responses, has been implicated in promoting tumor progression in various cancers, including breast cancer, as it increases cell proliferation and contributes to metastasis [46,47].

Therefore, the observed reduction in these cytokines following Delta infection hints at a potential oncolytic effect, mediated through cytokine modulation. However, this effect did not translate into significant changes in PDO morphology, as observed in the broader cancer PDO cohort. Nevertheless, this does not exclude the possibility of other cellular effects, such as the induction of cell death. This discrepancy suggests that the Delta variant may exert distinct biological impacts on breast cancer PDOs compared to other tumor types.

In contrast, the Gamma variant induced a significant reduction in PDO area and perimeter, accompanied by an increase in circularity, indicating a more pronounced morphological impact. Surprisingly, this morphological change was not associated with significant alterations in the levels of the measured cytokines. This divergence between morphological and cytokine responses highlights the complexity of viral interactions with cancer cells and suggests that different SARS-CoV-2 variants may exert distinct mechanisms of action other than immunological effects on breast cancer cells, highlighting the need for a deeper understanding of variant-specific oncolytic potential.

Overall, our results suggest that Gamma and Delta variants elicit distinct responses in breast cancer PDOs, likely reflecting tumor-intrinsic rather than systemic immune effects. The morphological changes observed with Gamma and the cytokine suppression induced by Delta underscore the complexity and variant specificity of SARS-CoV-2–tumor interactions.

In colorectal cancer PDOs, we observed distinct and variant-specific responses to SARS-CoV-2 infection. Organoids infected with the Delta variant showed reduced area and perimeter, suggesting a direct cytopathic effect. This was accompanied by a significant increase in MIP-1β release, a chemokine involved in immune cell recruitment and often associated with cellular stress or damage. The most circular organoids (0.9 bin) were exclusively from infected groups, both for Delta and Gamma, indicating a consistent morphological shift upon infection.

In contrast, Gamma-infected organoids from the same patients did not exhibit changes in area or perimeter. Instead, Gamma infection selectively increased E-selectin secretion, a molecule linked to endothelial adhesion and metastatic potential. These divergent patterns—Delta inducing structural disruption and MIP-1β release, and Gamma promoting E-selectin secretion—highlight distinct interactions between each variant and tumor cells.

This study presents some inherent limitations. PDOs were established from both primary and metastatic tumors, which differ in biological history and macroenvironmental context, factors that may influence the phenotypes observed. In addition, the limited number of patient samples and the restricted availability of biopsy-derived material constrained the scope of downstream analyses, precluding the inclusion of dedicated assays to directly quantify cell death or cytotoxicity. Future studies with expanded sample availability will be important to further dissect these mechanisms.

In conclusion, our study demonstrates that SARS-CoV-2 can exert an oncolytic effect in patient-derived tumor organoids and that this activity is dependent on both the viral variant and the tumor type. Gamma and Delta variants displayed distinct capacities to infect, replicate, and remodel the tumor microarchitecture, resulting in variant-specific morphological and immunological responses. These findings provide new insights for interpreting virus–tumor interactions and highlight the relevance of considering tumor-intrinsic features when evaluating the biological consequences of viral exposure in cancer settings. This work opens new perspectives for future investigations at the interface of oncology and virology.

## 4. Materials and Methods

### 4.1. Ethics Approval and Sample Collection

The current study was approved by the Research Ethics Committee of Grupo Oncoclínicas under registration number 5.812.518. Participants were cancer patients from Oncoclínicas & Co. (São Paulo, Brazil), undergoing treatment and follow-up between January 2022 and December 2023, who provided written informed consent to participate in this study. Inclusion criteria comprised patients aged ≥ 18 years diagnosed with stage IV metastatic breast, lung, or colorectal cancer. This study was conducted in accordance with the principles of the Declaration of Helsinki. Metastatic tumor tissue biopsies were obtained from stage IV patients with primary breast, lung, or colorectal cancers.

### 4.2. PDO Establishment and Culture

Fresh tumor tissue samples were used to generate PDOs. Establishment of organoid cultures was based on the protocol described by Zhao et al. [48], with minor modifications. Briefly, tissues were washed, finely minced, and subjected to mechanical and enzymatic dissociation. The resulting cell suspension was filtered through a 70 µm cell strainer, resuspended in a three-dimensional extracellular matrix, Matrigel (Corning, Corning, NY, USA), and seeded at a density of 1000 dissociated cells per well. After matrix polymerization, organoid culture medium based on Advanced DMEM/F12 (Gibco, Thermo Fisher Scientific, Waltham, MA, USA) was added, and cultures were maintained at 37 °C with 5% CO_2_.

Due to the intrinsic characteristics of each tumor, PDO formation occurred within 24 to 96 h after plating, with development regularly monitored via microscopy. Infections with SARS-CoV-2 variants were carried out within a 2- to 5-day window after PDO formation to ensure optimal culture maturity.

### 4.3. Viral Infection

The SARS-CoV-2 Gamma (P.1) and Delta (B.1.617.2) variants used in this study were kindly provided by Marilda Siqueira (Laboratory of Respiratory Viruses and Measles, IOC/Fiocruz). All viral stocks were expanded in Vero E6 cells and titrated by plaque-forming assay (PFU/mL) prior to use.

In a biosafety level 3 (BSL-3) laboratory, PDOs were infected with SARS-CoV-2 Gamma and Delta variants at a MOI of 0.01. The viral inoculum was prepared in a viral adsorption medium: RPMI-1640 (Gibco) supplemented with 2% fetal bovine serum (Gibco). The control group (NI—non-infected) received the vehicle. PDOs were incubated with the viral suspension for 1 h, with gentle agitation every 20 min to facilitate adsorption. After the adsorption phase, the PDOs were maintained at 37 °C in a humidified atmosphere containing 5% CO_2_ for 24 h. At 24 h post-infection, supernatants were collected for viral RNA extraction and cytokine quantification, while PDOs were harvested for whole-exome sequencing.

### 4.4. Microscopy

Images from PDOs were acquired using the EVOS XL Core microscope (ThermoFisher, Waltham, MA, USA). Images were captured at 0 h and 24 h post-infection with Gamma and Delta variants of SARS-CoV-2. The focal plane and magnification (40× objective lens) were kept consistent to ensure that images were acquired from the same wells at different time points, and analyses were performed at the population (pool) level, allowing for direct comparison before and after infection.

### 4.5. Shape Metric Analysis

Shape metrics, including area, perimeter, and circularity, were calculated from bright-field images of PDOs using ImageJ software [version 1.54g; National Institutes of Health (NIH), Bethesda, MD, USA]. PDOs were isolated from 8-bit grayscale images by adjusting the threshold, with manual adjustments applied as needed to precisely define each organoid’s boundaries.

The area and perimeter of each PDO were measured using ImageJ’s “Analyze Particles” tool, with area recorded in square pixels as the total selection area, and perimeter representing the length of the outer boundary of each PDO. Circularity was calculated using ImageJ’s formula: circularity = 4pi(area/perimeter^2^)

### 4.6. Real-Time Quantitative Polymerase Chain Reaction (RT-qPCR)

RNA was extracted from culture supernatants of infected and non-infected PDOs using the QIAamp Viral RNA Mini Kit (Cat. 52904, Qiagen, Venlo, The Netherlands) and quantified by one-step RT-qPCR with the SuperScript™ III Platinum™ One-Step qRT-PCR Kit w/ROX (Cat. 11745500, ThermoFisher). All samples were analyzed for SARS-CoV-2 N2 and human RNAse P targets using a commercial One-Step RTqPCR TaqMan kit, 2019-nCov CDC RUO Kit (IDT DNA, Cat. No. 10006625, Coralville, UA, USA), according to the manufacturer’s instructions. RTqPCR was performed in a 10 μL reaction containing 2 μL RNA, 5 μL Promega Go-Taq Probe-1-Step-RTqPCR System (2X) (Promega, Madison, WI, USA), 0.2 μL GoScript RT mix for 1-step RT-qPCR, 0.75 μL FAM/BHQ (N2 or RNAse P) TaqMan Primer-Probe Mix, 0.25 μL CXR Reference Dye (Promega), and 1.8 μL ultrapure water. Real-time PCRs were performed on the Applied Biosystems Quantstudio 3 Real-Time PCR System (ThermoFisher) using the following cycling conditions: 15 min at 50 °C, 10 min at 95 °C, followed by 40 cycles of 15 s at 95 °C and 60 s at 55 °C. Fluorescence was collected after each cycle in the annealing/extension step. All samples were run in duplicate, and the threshold was set to 0.02 in all analyses, on the QuantStudio™ Design & Analysis Software 2.7.0 (Applied Biosystems, Foster City, CA, USA). Viral load (copies/µL) was determined based on a standard curve generated from serial dilutions of known concentrations. Two samples (PDO_01 and PDO_12) were excluded from viral load analysis due to technical issues.

### 4.7. Cytokine Quantification

Supernatants from PDO cultures were collected 24 h after infection with the Gamma and Delta variants of SARS-CoV-2, as well as from the non-infected control group. The supernatants were used for cytokine quantification using the ProcartaPlex Multiplex Immunoassay kit (Cat. EPX200-12185-901, ThermoFisher). Each sample was probed for the following markers: E-selectin (CD62E), P-selectin (CD62P), GM-CSF, ICAM-1, IFN alpha, IFN gamma, IL-1 alpha, IL-1 beta, IL-10, IL-12p70, IL-13, IL-17A (CTLA-8), IL-4, IL-6, IL-8 (CXCL8), IP-10 (CXCL10), MCP-1 (CCL2), MIP-1 alpha (CCL3), MIP-1 beta (CCL4), and TNF alpha. Cytokine levels were quantitatively detected using the Luminex platform (ThermoFisher). Results were normalized for each patient’s non-infected control, and graphs are presented as fold change.

### 4.8. Statistical Analysis

All statistical analyses were performed using GraphPad Prism software (version 8.0.2; San Diego, CA, USA). For shape metrics analysis, outliers were identified and removed using the ROUT method (Q = 1%). As data did not follow a normal distribution (assessed by the Kolmogorov–Smirnov and D’Agostino–Pearson omnibus normality tests), non-parametric tests were applied. Comparisons among groups [NI(G), Gamma, NI(D), and Delta] were performed using the Kruskal–Wallis test, followed by Dunn’s multiple comparisons test for pairwise comparisons (NI(G) vs. Gamma, NI(D) vs. Delta, and NI(G) vs. NI(D)). Results are reported as median values with 95% confidence intervals (95% CI). For the quantification of immune mediators, group comparisons were performed using the Kruskal–Wallis test, followed by Dunn’s multiple comparisons test. Statistical significance was indicated as follows: * *p* < 0.05; ** *p* < 0.01; *** *p* < 0.001; **** *p* < 0.0001.

### 4.9. Whole-Exome Sequencing (WES) and Bioinformatics Analysis

Genomic DNA (gDNA) was extracted from PDOs using the AllPrep DNA/RNA Purification Kit (Cat. 80204, Qiagen). Sample preparation followed the Twist Exome 2.0 protocol (Cat. 104136, Twist Bioscience, South San Francisco, CA, USA) with minor adaptations. Briefly, DNA samples were fragmented for 9 min at 32 °C. Samples with concentrations below 1.25 ng/µL were concentrated using a speed vac for 30 min at 47 °C. A total of 10 ng of gDNA was used for target enrichment hybridization. WES was performed on the Illumina NextSeq 2000 platform using the NextSeq™ 2000 P3 cartridge (300 cycles, Cat. 20040561, Illumina, San Diego, CA, USA).

Data preprocessing adhered to the nf-core best practices and pipeline (nf-core/sarek v.3.2.3) [49,50]. Somatic variant calling was performed using Mutect2 and Strelka2. Only the variants called by both tools were considered. A filtered VCF file was generated from the raw VCF data by applying the following thresholds: allele frequency (AF) ≥ 5%, read depth (DP) ≥ 5, median mapping quality (MMQ) ≥ 40, and global minor allele frequency (GMAF) ≤ 1%. Data analysis was performed using the maftools package (Bioconductor in R; version 4.4.0; Vienna, Austria) [51,52].

## Figures and Tables

**Table 1:** Patient and biopsy details: cancer type, sample ID (PDO_#), age (years), gender, and biopsy site.

Cancer Type	Sample ID	Age	Gender	Biopsy Site
Lung	PDO_01	29	Male	Metastatic
	PDO_04	82	Female	Metastatic
	PDO_05	74	Male	Primary
	PDO_08	80	Male	Primary
	PDO_12	69	Male	Metastatic
	PDO_16	58	Female	Metastatic
	PDO_17	59	Male	Metastatic
	PDO_18	46	Female	Metastatic
Breast	PDO_06	45	Female	Metastatic
	PDO_07	48	Female	Metastatic
	PDO_09	50	Female	Primary
	PDO_13	61	Female	Metastatic
Colorectal	PDO_02	35	Male	Metastatic
	PDO_03	63	Male	Metastatic
	PDO_11	61	Male	Metastatic
	PDO_14	62	Male	Primary
	PDO_15	66	Male	Metastatic

**Table 2:** Somatic nonsynonymous mutations in viral infection-related genes.

Gene	PDO	Consequence	Position	Ref	Alt	Amino acid change	Function
*DAAM1*	PDO_18	missense variant	59,340,156	A	C	Q/P	actin and small GTPase binding
*DNMT1*	PDO_16	upstream gene variant	10,195,044	G	A		DNA and lncRNA binding, DNA-methyltransferase activity
*FOXA2*	PDO_07	missense variant	22,582,785	C	T	D/N	DNA binding, transcription corepressor activity, chromatin organization, positive regulation of cell-cell adhesion mediated by cadherin, negative regulation of epithelial to mesenchymal transition
*FYCO1*	PDO_05	missense variant	45,967,167	C	A	A/S	microtubule plus-end-directed vesicle transport mediator
*IFNAR1*	PDO_15	missense variant	33,355,347	T	A	L/Q	cytokine binding, JAK pathway signal transduction
*IL6*	PDO_15	Intron variant	22,729,769	G	A		growth factor activity, acute-phase response, cell surface receptor signaling pathway via JAK-STAT
*LRRC15*	PDO_09	missense variant	194,359,788	C	T	R/Q	collagen, fibronectin and laminin binding, receptor-mediated virion attachment to host cell
*LRRC15*	PDO_15	missense variant	194,360,491	G	A	L/F	collagen, fibronectin and laminin binding, receptor-mediated virion attachment to host cell
*MUC5B*	PDO_17	missense variant	1,250,198	C	G	P/A	major component of mucus secretions, metal ion binding
*RAB7A*	PDO_05	3 prime UTR variant	128,814,253	A	C		G protein activity, small GTPase binding, endocytosis, intracellular transport
*RAB7A*	PDO_03	3 prime UTR variant	128,814,253	A	C		G protein activity, small GTPase binding, endocytosis, intracellular transport
*SFTPD*	PDO_07	missense variant	79,942,068	C	T	E/K	macrophage chemotaxis, regulation of phagocytosis, regulation of cytokine production
*SLC6A20*	PDO_16	missense variant	45,759,072	A	G	L/P	amino acid transmembrane transporter activity
*STAT3*	PDO_02	missense variant	42,323,019	C	T	E/K	signal transducer and transcription activator that mediates cellular responses to interleukins and other growth factors, DNA and lncRNA binding
*TET2*	PDO_02	missense variant	105,276,472	C	A	P/T	DNA demethylation, DNA binding
*TLR3*	PDO_15	missense variant	186,083,880	T	C	F/L	component of innate and adaptive immunity, control of host immune response against pathogens through recognition of molecular patterns specific to microorganisms
*TPCN2*	PDO_18	missense variant	69,055,254	G	A	V/M	calcium channel activity, ligand-gated sodium channel activity, endolysosomal trafficking

## Data Availability

The data presented in this study are available on request from the corresponding author. The data are not publicly available due to privacy or ethical restrictions.

## Abbreviations

The following abbreviation is used in this manuscript:

ROUT	Robust regression and outlier removal

## Appendix A

**Table A1:** SARS-CoV-2 infection-related gene list.

ABO	CCL5	DPP4	IFITM1	IRF9	PPIA	TNF
ACE2	CCR9	DPP9	IFITM2	ISG15	RAB7A	TPCN2
ADAM17	CTSB	EMMPRIN	IFITM3	JAK1	SFTPD	TYK2
AHSG	CTSL	FOXA2	IFNAR1	LRRC15	SLC6A20	XBP1
ANPEP	CXCL10	FURIN	IFNAR2	LZFTL1	STAT1	XCR1
AP2M1	CXCL8	FYCO1	IFNG	MIF	STAT3	
APO3	CXCR6	GATA6	IFNL1	MX1	TET2	
ATF4	DAAM1	HDAC	IL1B	NRP1	TLR3	
BSG	DDIT3	HFN1A	IL6	OAS1	TLR7	
CCL2	DDX58	HLA-A	IRF3	PAICS	TMPRSS2	
CCL20	DNMT1	HLA-B	IRF7	PIKFYVE	TMPRSS4	
